# A synthetic data generation system for myalgic encephalomyelitis/chronic fatigue syndrome questionnaires

**DOI:** 10.1038/s41598-023-40364-6

**Published:** 2023-08-31

**Authors:** Marcos Lacasa, Ferran Prados, José Alegre, Jordi Casas-Roma

**Affiliations:** 1 ADaS Lab - E-Health Center, Universitat Oberta de Catalunya, Rambla del Poblenou, 156, 08018 Barcelona, Spain; 2 Center for Medical Image Computing, University College London, London, UK; 3 National Institute for Health Research Biomedical Research Centre at UCL and UCLH, London, UK; 4 Department of Neuroinflammation, Queen Square MS Center, UCL Institute of Neurology, Faculty of Brain Sciences, University College London, London, UK; 5 ME/CFS Unit, Division of Rheumatology, Vall d’Hebron Hospital Research Institute Universitat Autònoma de Barcelona, Barcelona, Spain

**Keywords:** Computational models, Data acquisition

## Abstract

Artificial intelligence or machine-learning-based models have proven useful for better understanding various diseases in all areas of health science. Myalgic Encephalomyelitis or chronic fatigue syndrome (ME/CFS) lacks objective diagnostic tests. Some validated questionnaires are used for diagnosis and assessment of disease progression. The availability of a sufficiently large database of these questionnaires facilitates research into new models that can predict profiles that help to understand the etiology of the disease. A synthetic data generator provides the scientific community with databases that preserve the statistical properties of the original, free of legal restrictions, for use in research and education. The initial databases came from the Vall Hebron Hospital Specialized Unit in Barcelona, Spain. 2522 patients diagnosed with ME/CFS were analyzed. Their answers to questionnaires related to the symptoms of this complex disease were used as training datasets. They have been fed for deep learning algorithms that provide models with high accuracy [0.69–0.81]. The final model requires SF-36 responses and returns responses from HAD, SCL-90R, FIS8, FIS40, and PSQI questionnaires. A highly reliable and easy-to-use synthetic data generator is offered for research and educational use in this disease, for which there is currently no approved treatment.

## Introduction

Myalgic encephalomyelitis, commonly called chronic fatigue syndrome (ME/CFS), is a serious, complex, and chronic multisystem illness of unknown etiology, often triggered by a persistent viral infection (for this reason, it is also known as post-viral fatigue syndrome). ME/CFS affects as many as 17 to 24 million people worldwide, and its prevalence is expected to double by 2030^1^. It is characterized by unexplained and persistent post-exertional fatigue that is not relieved by rest. It is exacerbated by physical and mental exertion and other core symptoms such as cognitive, immunometabolic, autonomic, and neuroendocrine dysfunction^2^. It produces severe disability in patients, significantly interfering with their work activity and their daily life tasks^3^. In addition to fatigue, these patients have characteristic inflammatory and muscular symptoms, sleep dysfunction, and altered cognitive functions^4^. The symptomatic muscle blocks symptoms such as pain, generalized muscle weakness, fatigue after physical exertion, neurological symptoms (sensory hypersensitivity, ataxia, dysmetria, visual disturbances, and motor incoordination), neurocognitive symptoms (alterations in memory, concentration, calculation, task planning). The autonomic block (cephalic instability, dizziness, fainting spells, excessive sweating, orthostatic hypotension, tremor or alterations in intestinal rhythm), immunoinflammatory symptoms (low-grade fever, sore throat, recurrent canker sores, polyarthralgia, morning numbness, infections such as herpes or candida) and deficiency symptoms in the production of cellular metabolic energy. Sleep disturbances have been relevant since their description as their clinical entity. In all versions of the different ME/CFS diagnostic criteria, sleep disorders have played a key role, especially the presence of unrefreshing sleep and the importance of the Pittsburgh Sleep Quality Index (PSQI) questionnaire in the assessment of the severity of alterations in sleep quality and its association with fatigue, pain, psychopathology, and neurovegetative dysfunction^5^. ME/CFS, together with the symptomatic complexity that it presents, as a consequence of its multisystemic nature, is associated with different comorbid phenomena such as fibromyalgia, sicca syndrome, myofascial syndrome, psychopathology, ligament hyperlaxity, fasciitis plantar, degenerative vertebral disease or mechanical, shoulder tendinopathy, multiple chemical sensitivity, epicondylitis, carpal tunnel syndrome, osteoporosis, hypercholesterolemia, hypertriglyceridemia, vascular risk, endometriosis, thyroiditis, with a higher prevalence than that observed in patients not affected by ME/CFS^6^.

In the study of ME/CFS, after the diagnosis and assessment of comorbid phenomena, it is essential to quantify and assess fatigue, quality of life, or anxiety/depression psychopathology using a battery of clinically self-administered questionnaires. Today there are few units specialized in ME/CFS in the world, with a relatively low number of duly documented cases and a lack of publicly available data compared with other disorders. Moreover, unfortunately, there are no commercially available diagnostic tests, no specific lab biomarkers, and no targeted FDA-approved drugs for ME/CFS^7^. Therefore, each subject to be diagnosed with ME/CFS must undergo a Fukuda criteria evaluation and procedure that each unit has established using batteries of validated self-administered questionnaires. As stated before, it is important to evaluate the disabling fatigue perception, sleep problems, and health-related quality of life using self-administered questionnaires such as the fatigue impact scale FIS40^8^ and FIS8^9^, PSQI^10^, and Short Form Health Survey (SF-36)^11^, Symptom Checklist-90-revised (SCL 90 R) psychological inventory^12^, hospital anxiety and depression scale (HAD)^13^. Ongoing placebo-controlled clinical trials to evaluate the clinical benefits of drugs on ME/CFS symptoms^14^ have changed some questionnaire scores from baseline to final study as a primary endpoint.

There is no consensus on the number and type of questionnaires that should be carried out, so not all units record the same number per subject. Consequently, it is complex for ME/CFS units to have many records of the questionnaires necessary to efficiently approach large longitudinal and multicenter studies of patients with this pathology using the latest advances in data analysis, such as Machine Learning techniques.

Machine Learning is a particular method of data analytics that automates model building as it relates to the development of models. Over the last years, it has been proven great performance of machine learning supervised algorithms in several clinical applications^15^ to diagnose and treat diseases. Supervised learning involves training machine learning-based algorithms using labeled input datasets requiring however, to be efficient and get optimal results, a large number of records are needed. The learning occurs by comparing results with the expected outputs to identify errors and change the model's weights to infer knowledge. There are few publications, and all of them very recent, that refers to the application of different machine learning techniques in ME/CFS: seeking a new biomarker^16^, clustering^17^, or discovering the relationship between depression and ME/CFS^18^, using neural networks seeking omic biomarkers^16^ or neural networks classifiers^19^. While they all make important steps forward in understanding ME/CSF, the limited sample size makes generalization and translation of their findings to clinical practice or other datasets difficult. Also, as stated before, when there are no clear biomarkers to follow the evolution of the illness, like in ME/CSF, quality-of-life questionnaires are used to measure it^14^. There are several lines of investigation, such as clustering^20^ or finding relations between blood measurements with questionnaires data^21^.

Therefore, there is an increasing demand to access large repositories of high-quality health datasets for better and more reliable predictions from supervised machine learning algorithms. Anonymized electronic health records are bought and sold by insurance^22^ and clinical groups^23^. However, they are limited in size or content, might be incomplete, and their applications might be restricted. This problem can be overcome using synthetic datasets coming from simulations^24,25^. Synthetic datasets are generated to create data for improving the sample size of existing cohorts or filling in the missing values, preserving privacy while keeping the real data characteristics. Synthetic data generators preserve the statistical properties of the original. However, they do not reveal any information regarding real people and offer several benefits, such as overcoming real data usage restrictions of data sharing and patient consent. There is a need for developing synthetic datasets that would complement real-world data for various reasons^26^: ease of access, cost-efficiency, test-efficiency, patient privacy protection, completeness, and validation capabilities, handling missingness, complex interactions between variables, resulting sensitivity analysis statistics from latest classifiers and graphical modeling and resampling^27^. A common application of synthetic data generation in medicine is image generation simulating diseases. It helps to test and benchmark the performance and accuracy of different algorithms. Some recent applications are in the simulation of skin lesions^28^, brain atrophy in aging or Dementia^29^, generation of PET MRI scans for Alzheimer's disease^30^, tumor generation in the brain^31^, or breast cancer^32^.

This work aims to generate a robust and reliable synthetic data generator for ME/CFS questionnaires to produce high-fidelity and risk-free health care records, enhance existing public and private ME/CFS datasets for investigation and educational use, and are free of legal, privacy, security, and intellectual property restrictions.

## Patients and methods

### Dataset

This prospective cross-sectional study includes 2,522 subjects diagnosed with ME/CFS from the Vall d’Hebron University Hospital, Barcelona, Spain, 90.5% females (mean age 48.11 ± 10.31 years) and 9.5% males (mean age 44.41 ± 11.35 years). Data for SF-36, HAD, FIS8, FIS40, SCL 90 R, and PSQI questionnaires has been obtained and recorded from 2008 to 2021. See Table 1 for final records. Patients were eligible to participate if they were 18 years, had a confirmed diagnosis of ME/CFS, met the Fukuda^33^ and Carruthers criteria^34^, and provided signed written informed consent and ethics committee approval. The data collected were anonymized in a database to which only those designated for the study had access, and in no case was any information known that could reveal or infer the participant’s identity.

**Table 1:** Available data for each questionnaire and the questionnaires' characteristics.

Questionnaire	Registers	Questions	Subscales	Total value	Answers’ rank
SF 36	2346	36	10	NO	{1,2,3,4,5,6}
HAD	2339	14	2	YES	{0,1,2,3}
FIS8	2057	8	0	YES	{0,1,2,3,4}
FIS40	2362	40	3	YES	{0,1,2,3,4}
SCL 90 R	2361	90	12	NO	{0,1,2,3,4}
PSQI	1959	34	7	YES	{0,1,2,3}

From left to right, each column title means Registers: number of available forms. Questions: number of questions per questionnaire. Subscales: number of defined subscales. Total Value: The questionnaire has a unique resume value. Answers’ rank: Possible answer value for each question.

### Relationship graph between questionnaires

Graph theory was used to analyze the relationships between the subscales of each questionnaire. A graph is a collection of nodes (also called vertices) joined together in pairs by edges (undirected) or arcs (directed)^35^. The graph structure allows us to capture the pattern of interactions between the nodes (individuals or entities). Graph (or network) analysis is used to study relationships between individuals to discover knowledge about global and local structures. The study of structure networks helps to decide the optimal order^36^.

In this work, the graph nodes are defined as all subscales, and the edges are defined as moderate or strong correlations between nodes (subscales). The linear correlation between two subscales is represented by $corr\left(i,j\right)$, and Pearson correlation is defined as moderate or strong if $corr\left(i,j\right)\ge 0.5$ ^37^ in case of direct correlation. An $edge\left(i,j\right)$ is defined if $abs\left(corr\left(i,j\right)\right)\ge 0.5$.

The relation of subscales between each test is related in Table 2. Each subscale has been classified according to the area to which it has been defined and named as the subject. Thirty-eight subscales, six tests, and twelve subjects form the dataset to create the relationship between them to the graph.

**Table 2:** Subscales and subject definitions for questionnaires.

Test	Subscale	Subject
SF-36	Physic function (PF)	Physic
	Rol physic (RP)	Physic
	Body pain (BP)	Pain
	General health (GH)	General health
	Vitality (VT)	Vitality
	Social function (SF)	Social
	Rol emotional (RE)	Emotional
	Mental health (MH)	Mental
	Physical component score (PCS)	Physic
	Mental component score (MCS)	Mental
HAD	Total anxiety	Anxiety
	Total depression	Depression
	Total HAD	Depression
FIS40	Physic dim	Physic
	Cognitive dim	Cognitive
	Social dim	Social
	Total FIS40	Physic
FIS8	FIS8	Physic
PSQI	Component 1	Sleep quality
	Component 2	Sleep quality
	Component 3	Sleep quality
	Component 4	Sleep quality
	Component 5	Sleep quality
	Component 6	Sleep quality
	Component 7	Sleep quality
	Total PSQI	Sleep quality
SCL 90 R	Somatizations (SOM)	Mental
	Obsessions (OBS)	Mental
	Interpersonal sensitivity (SI)	Mental
	Depression (DEP)	Depression
	Anxiety (ANS)	Anxiety
	Hostility (HOS)	Anxiety
	Phobic anxiety (FOB)	Anxiety
	Paranoid (PAR)	Mental
	Psychoticism (SIC)	Mental
	Severity global index (GSI)	Mental
	Positive symptoms (PST)	Mental
	Symptomatic discomfort Index (PSDI)	Mental

Test: Each of the analyzed questionnaires. Subscales: Every dimension defined in every questionnaire. Subject: Area that is associated with each subscale.

The study of the relationships mentioned above should indicate the order to generate our machine learning models. The SF-36 is prevalent and will be used in our model as initial data. The rest of the order will be given by the relationships between the different tests so that those with a stronger relationship are consecutive in the model. The strength of the relationship is measured in terms of the percentage of connections between the test nodes.

$$max\,\, (re{l}_{i}/re{l}_{j})\,\, for\,\, each\,\, i, j \forall i,j \in [1,n] in\, n \,test$$

$$re{l}_{i} = number\,\, of\, \,nodes\, \,of\,\, test\, \,i\, \,related\, \,with\, \,nodes\, \,of\, \,test\, \,j$$

$$re{l}_{j} = number\, \,of\, \,nodes\, \,of\, \,test\, \,j$$

### Model architecture

**Figure 1:**
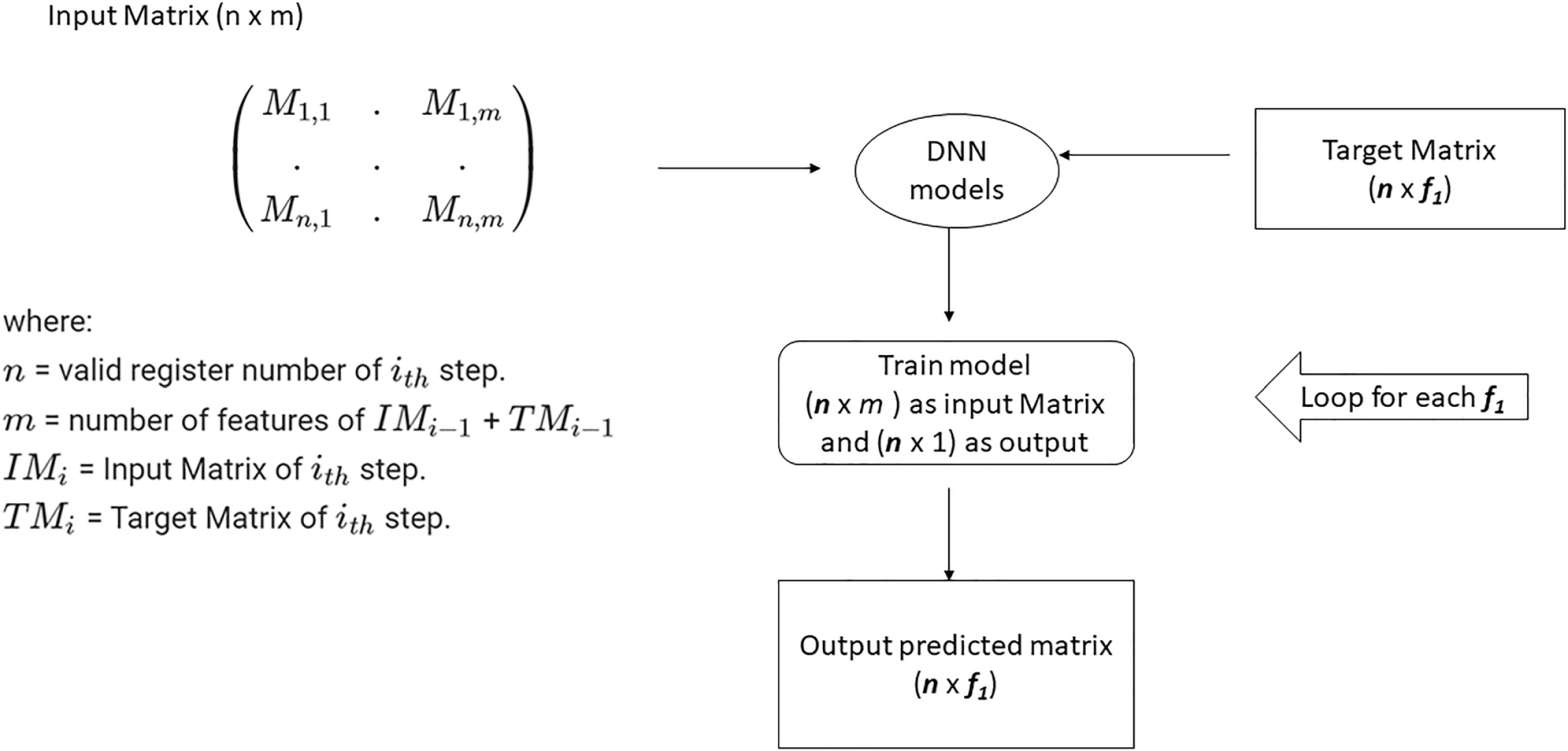
Modeling schema. The model requires a questionnaire answers matrix as an input value. The output is the other five questionnaires. Each questionnaire has a different number of questions and subscales. A simple sum of the number of questions calculates most subscales. For example, the SF-36 input dimension is n × 36, where n is the number of patients who answered the SF-36 questionnaire. The output is n × 186, where 186 is all five questionnaire answers.

Real data of all of six questionnaires are required to train and build the models. First, an input matrix represents the validated answers of a number of patients, where *n* is the number of validated responses and *f* the number of questions. That is the first training data. As predicted, it has to be a second questionnaire which the same *n* and *f*_1_ questions. The model must generate a predicted matrix with the same dimension. The next step has as input matrix the initial matrix concatenated with the last predicted matrix and the second questionnaire response matrix for prediction, as shown in Fig. 1.

### Machine learning algorithms

Classification and regression models can be used. The goal is to provide 186 output dimensions that must be calculated step by step. The output is compared with the real data set to validate the model. The results are validated using the t-student test. The strategy is to validate one questionnaire. The next step is concatenating the questionnaire answers matrix as input with the final output with different models. It has tested machine learning and deep learning algorithms step by step. The validation system has measured whether real and synthetic data come from the same populations within t-student statistics. The models tested have been regressors and classifiers. The comparison between XGBoost and Deep Neural Networks (DNN)^38,39^ shows that both models offer similar performance in structured data.

### Validation metrics

The F1-score can be interpreted as a harmonic mean of precision and recall, where an F1-score reaches its best value at one and worst score at zero. The relative contribution of precision and recall to the F1-score are equal. The formula for the F1 score is:

$${F}_{\beta }=\left(1+{\beta }^{2}\right)\frac{precision\times recall}{{\beta }^{2}\,precision+recall}$$

and operating,

$$F1=\frac{TP}{TP+\left(FN+FP\right)}$$

where TP is the number of true positives, FN is the number of false negatives, and FP is the number of false positives. Better performance means lower FN and FP values, and better precision and recall mean better F1 performance. In imbalanced data, greater accuracy than the F1 score indicates that some labels perform poorly. Recall is defined by the ratio $recall=\frac{tp}{tp+fn}$^40^. Accuracy is defined if $\left(y,\widehat{y}\right)$ as a (sample, predicted), then the fraction of correct predictions over samples is defined as

$$accuracy\left( {y,\hat{y}} \right) = \frac{1}{{n_{{samples}} }}\sum\limits_{{i = 0}}^{{n_{{samples}} - 1}} {1\left( {\widehat{{y_{i} }} = y_{i} } \right)}$$

Mean error is defined as the ratio of overall value questionnaire predicted versus comprehensive value sample questionnaire. The series for t-student value is defined as the sum of all answers of each questionnaire variable, predicted, and sample data.

### Ethics approval

The authors declare that the procedures followed were by the regulations of the responsible Clinical Research Ethics Committee and by those of the World Medical Association and the Helsinki Declaration. The research protocols were approved by the Ethics Committee of the Vall d'Hebron University Hospital, the first “Population-based Registry of Patients with Chronic Fatigue Syndrome” approved on 18/10/2006.

## Results

### Relationship across questionnaires

**Figure 2:**
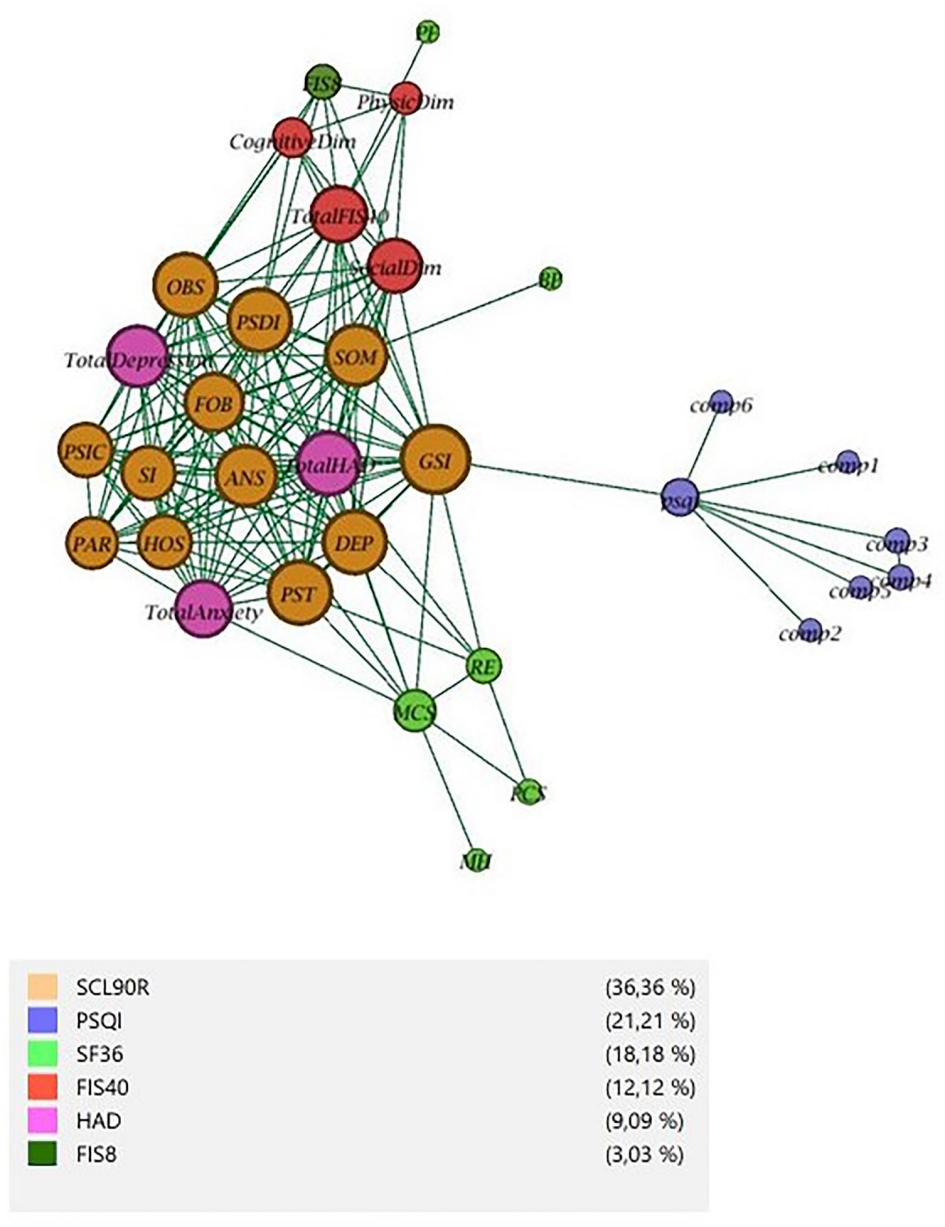
Subscales relationship graph. Color nodes represent the test to which nodes belong. The percentage in the legend represents the number of nodes versus the total*.*

In our proposed model, an $edge\left(i,j\right)$ is defined if $abs\left(corr\left(i,j\right)\right)\ge 0.5$ which indicates moderate or strong direct and indirect correlation. The 2370 registers were validated, and the Pearson correlation analyzed 38 questionnaire subscales. The subject of each subscale represents networks with each node (9) shown in Fig. 2. Mental, depression, and anxiety are strongly correlated with physical subjects. SF-36 emotional subscales are relational with anxiety, depression, and mental subscales (SCL 90 R and HAD questionnaires). As can be seen, HAD and SCL 90 R are strongly correlated. The node's size is related to the degree of the node, i.e. the number of incident edges.

In supplementary material, the second network analyzes the subscales as a node and the same relationship as an edge. The SF-36 subscales (green) have strong relationships with HAD (magenta) and FIS8, and FIS40 (strong-green and red, respectively). SCL 90 R (brown) has a strong relationship with HAD. Furthermore, PSQI (blue) has no relationship except the total psqi value. The strength of the relationship is measured in terms of the percentage of relationships between the test nodes. The initial test is SF-36, and its nodes have relationships with 100% of HAD’s nodes (3 of 3) and only 25% of SCL 90 R (4 of 12). HAD’s nodes have a 100% relationship with SCL 90 R’s nodes. SCL 90 R has a relationship with the unique FIS8 node, which has relationships with all four FIS40 nodes. The last test with few relations is PSQI. Consequently, the order decided according to aforementioned relationships is: HAD, SCL 90 R, FIS8, FIS40, and PSQI.

### Best model selection

A test comparison between XGBoost, Classifier and XGBoost Regressor using SF-36 as training data and HAD as a target with 2321 validated registers, is provided in supplementary material Fig. 3. The hyperparameter defines how our model works^41^. The parameters tuned were *max_depth, gamma, reg_alpha, reg_lambda, colsample_bytree, min_child_weight, subsample, n_estimators* and *eta*. Hyperopt has been used for hyperparameter tuning^41^. Both must be trained for each question; therefore 14 models have to be trained. The order on a set predicted value is {0, 1, 2, 3}, and the trained value is {1, 2, 3}, where in both cases, greater values show worse health status. Regressor predicted rounded to compare between real data. The results of the model are analyzed with XGBoost and the regression and classification are compared. The mean regression error is much higher than the classification error (32.50% vs. 3.16%). Therefore, the regression model is discarded in the following analyses (the results are available in the supplementary material, Table S1). Total connections have been 32,494 (2321 registers × 14 questions HAD questionnaire) and “1” and “2” answers are 67.25% of the total. The model tends to reduce the mean error, so the model predicted 70% more “1” than real and rare predicted, “3” (For more information, see Table S3 in the Supplementary Material).

Imbalanced data occur where one or more class labels have a very high number of observations, and the other has a lower one. The main problem is to increase accurate predictions of the minority class. To consider the skewed distribution of classes of different weights, classes with weights result in a penalty and a minor update of the model coefficients. The model based on the Keras library is more flexible, and for each question, it can be considered as the difference of the unbalanced data. The main difference between the Keras classifier model is the usage of the recall value, which helps to reduce the aforementioned problem with imbalanced data (for more information, see Table S3 in the Supplementary Material). For each class, do

$$classWeigh{t}_{i}=\frac{n}{\left(classes\times coun{t}_{i}\right)}$$

where *n* is the number of valid registers, *classes* is the number of classes, and $coun{t}_{i}$ is the support of *i*th class. Results comparison 1st questions of HAD (for more information, see Table S1–S3 in the Supplementary Material). The answer “0” has 66 (2.8%) support, and the answer “3” has 562 (24.21%) support. Minority-weighted label classes tend to be underrepresented with a low recall rate, 0.00 in the first case. These biases produce worse synthetic quality data for posterior analysis. Table 3 shows the results once corrected by the configuration in our model, improving the results significantly in those responses with low representation.

**Table 3:** Keras weighted model results.

Answers	Precision	Recall	f1-score	Support	Class weights
0	0.62	0.79	0.69	66	8.80
1	0.71	0.83	0.76	886	0.66
2	0.66	0.59	0.62	807	0.72
3	0.83	0.69	0.75	562	1.03
Accuracy			0.71	2321	
Macro avg	0.70	0.73	0.71	2321	
Weighted avg	0.72	0.71	0.71	2321	

### Model results

**Figure 3:**
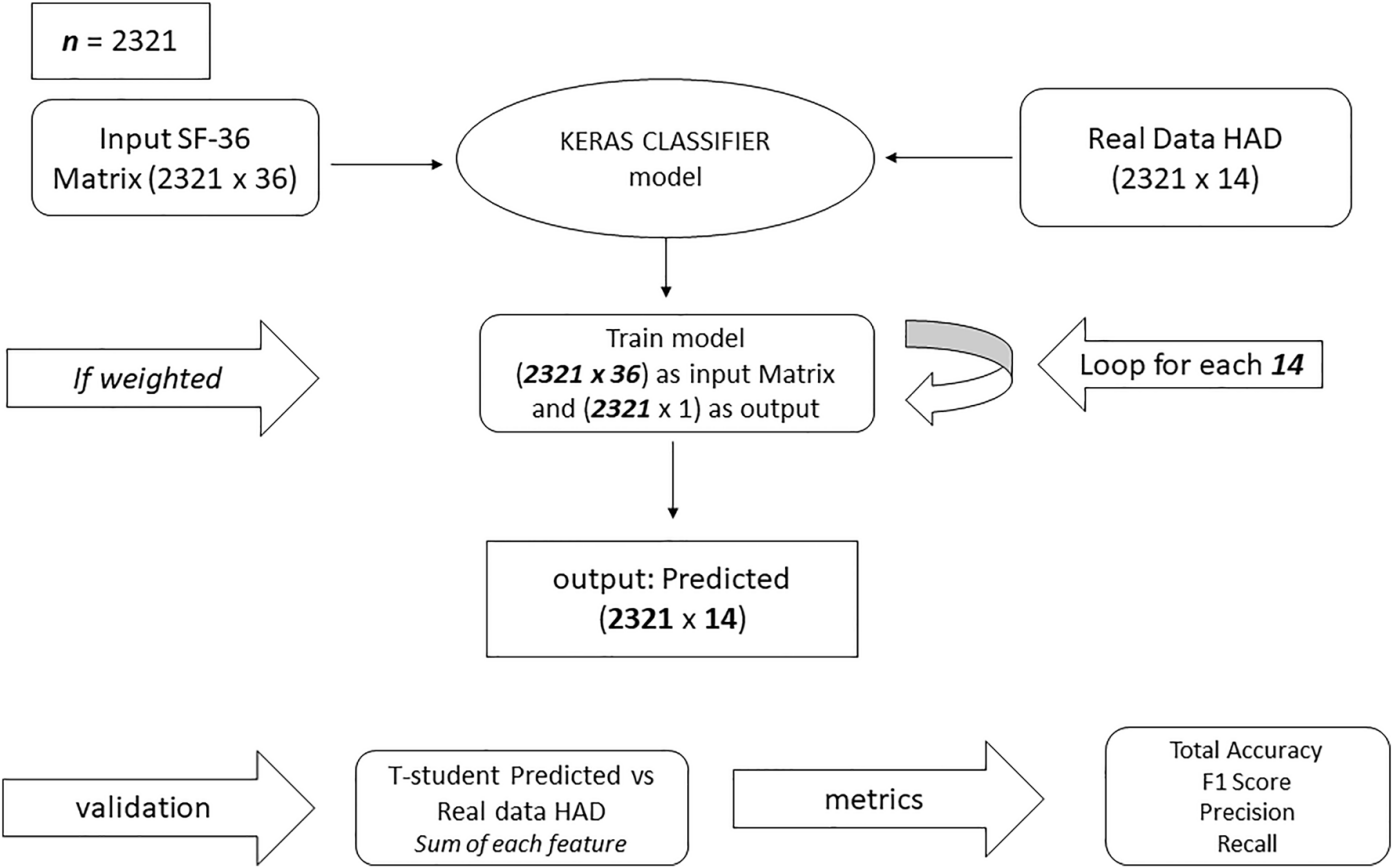
Keras Classifier algorithm schema.

Building the model needs five steps, as depicted in Fig. 3. The first step requires an SF-36 questionnaire input matrix with 3019 registers which HAD questionnaire had the same. The output is a HAD synthetic matrix. The second step requires an input matrix of SF-36 + HAD (synthetic data) and produces synthetic SCL 90 R responses and so on. The results are detailed in Tables 4, 5.

**Table 4:** Final model result.

Metrics	Steps models summary				
	STEP 1	STEP 2	STEP 3	STEP 4	STEP 5
Inputs models (dimension)	SF-36 (2321 × 36)	SF-36 + HAD (2314 × 50)	SF-36 + HAD + SCL 90 R (2019 × 140)	SF36 + HAD + SCL 90 R + FIS8 (2019 × 148)	SF-36 + HAD + SCL 90 R + FIS8 + FIS40 (1902 × 188)
Accuracy	0.67	0.78	0.81	0.78	0.78
Precision	0.69	0.78	0.83	0.79	0.80
Recall	0.72	0.76	0.76	0.74	0.76
F1 score	0.70	0.77	0.81	0.76	0.78
Mean error	− 1.35%	− 1.22%	− 2.59%	− 2.81%	− 5.50%
t-student	0.79	0.85	0.67	0.18	0.37
output	HAD	SCL 90 R	FIS8	FIS40	PSQI

Each question of each step needs different parameters, so it has to train 188 models with other parameters.

**Table 5:** Steps models summary.

Inputs models (dimension)	SF-36 (2321 × 36)	SF-36 + HAD (2314 × 50)	SF-36 + HAD + SCL 90 R (2019 × 140)	SF36 + HAD + SCL 90 R + FIS8 (2019 × 148)	SF-36 + HAD + SCL 90 R + FIS8 + FIS40 (1902 × 188)
Layers	4	3	4	4	4
Dropout	2	2	3	3	2
epochs	4000	3000	3000	3000	3000
Monitor	*Val_recall*	*Val_recall*	*Val_recall*	*Val_recall*	*Val_recall*
Early stopping patience	400	300	300	300	400
Neurons [layer]	[400,400,200,100]	[1500,1500,750]	[1000,1000,500,250]	[1000,1000,500,250]	[500,500,250, 100]
output	HAD	SCL 90 R	FIS8	FIS40	PSQI

## Discussion

Given the SF-36 questionnaire data can create using a new model, synthetic responses from other questionnaires inform the impact of fatigue, psychological phenomena, and sleep dysfunction. The lack of risk-free health data is an issue in ME/SFC hospital units and investigators. This open-source project offers a tool to generate risk-free synthetic data for the health IT and clinical community to use, experiment, and create more synthetic data. The quality based on validation tests did not cover projects or research focused on clinical discovery. Synthetic data can be an alternative to ground truth when data access is restricted and an excellent alternative to machine learning training/testing datasets^26^.

The SF-36 includes one multi-item scale that assesses eight health concepts: (1) limitations in physical activities because of health problems; (2) limitations in social activities because of physical or emotional problems; (3) limitations in usual role activities because of physical health problems; (4) bodily pain; (5) general mental health (psychological distress and well-being); (6) limitations in usual role activities because of emotional problems; (7) vitality (energy and fatigue), and (8) general health perceptions and is one of the most used quality life questionnaires used and evaluated^41^. The other five questionnaires used in this work complement most information about the quality of life of ME/SFC patients.

The questionnaires can be answered quickly and are regularly available in primary care and specialized medical consultations. Some applications offer automated analyzed results that inform essential information about patient health conditions.

The graph theory has been used to decide the order of the modeling cascade. Although a deeper analysis of these relationships should be the subject of another, more specific work, in this case, it informs us of the order used in our model. These relationships will characterize our model, which will be more robust with more records analyzed. Our dataset is unusually great in SFC, which becomes robust to our models.

Our synthetic dataset generator applications fill in missing data of real datasets from any other five questionnaires. For those, ME/SFC dataset clinical units with SF-36 questionnaire answers but missing others could build a complete dataset.

## Limitations

(1) Single-center trial. (2) Unit of reference in diagnosing and treating CFS/ME, which may be biased towards more severe cases and a longer evolution time than studies in primary care. (3) No information is available on parameters such as the results of the two-day ergometric test for assessing exercise intolerance, a neuropsychological battery for assessing cognitive impairment, and neurovegetative dysfunction, e.g., heart rate variability. (4) That this is a prospective study with cross-sectional data collection. It is not a longitudinal study.

## Conclusion

Synthetic patients can be simulated with models of ME/CFS questionnaires data and corresponding standards of care to produce risk-free realistic synthetic healthcare records at scale. An open-source generator offers high-fidelity synthetic data for investigation and educational use, free of legal, privacy, security, and intellectual property restrictions.

### Supplementary Information

Supplementary Information.

## Data Availability

GitHub is an online platform where researchers and software developers share their work with the scientific community. The following link shares the work described here. The datasets generated and/or analyzed during the current study are available in the SFCSyntheticDataGenerator repository, https://github.com/mlacasa/SFCSyntheticDataGenerator
