# Supplementary material for: A synthetic data generation system for myalgic encephalomyelitis/chronic fatigue syndrome questionnaires
Source: Sci Rep. 2023 Aug 31;13:14256. doi: 10.1038/s41598-023-40364-6 (PMC10471690; doi:10.1038/s41598-023-40364-6)
Supplement: Supplementary file 1 — Supplementary Information. [file 41598_2023_40364_MOESM1_ESM.docx]

## SUPPLEMENTARY MATERIAL

**Figure S1**.- *The components of Table 4 are analyzed according to the subject, being part of the different questionnaires analyzed. The size of the node depends on its degree.*


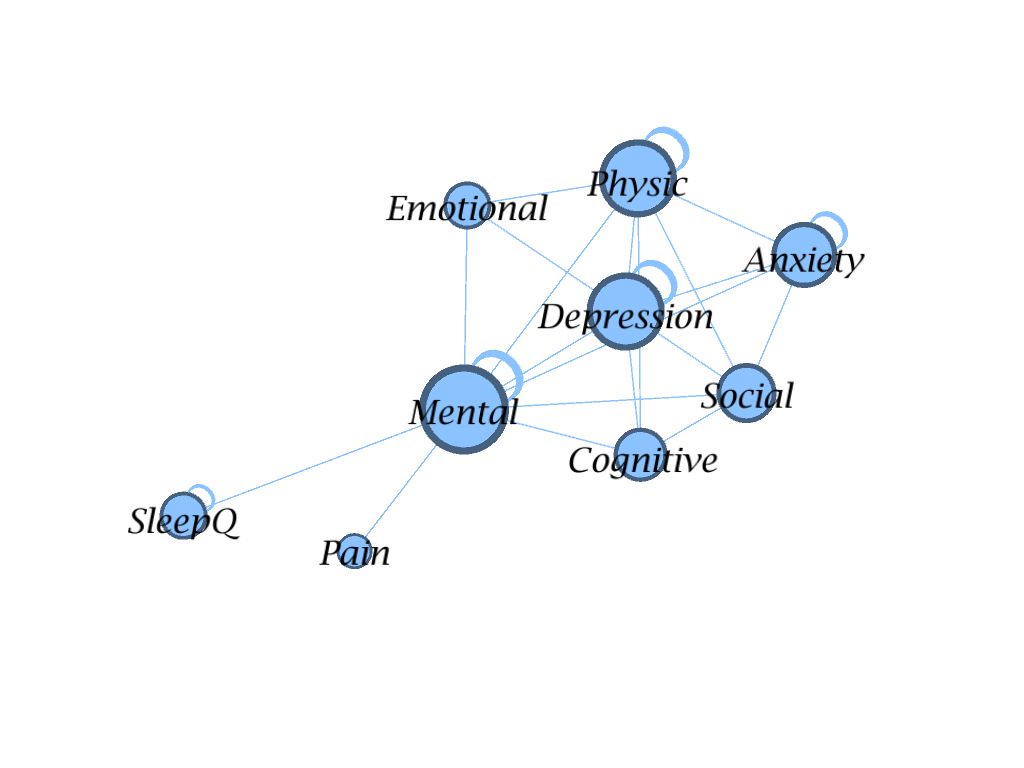


**Figure S2. -** Algorithm XGBoost model schema.


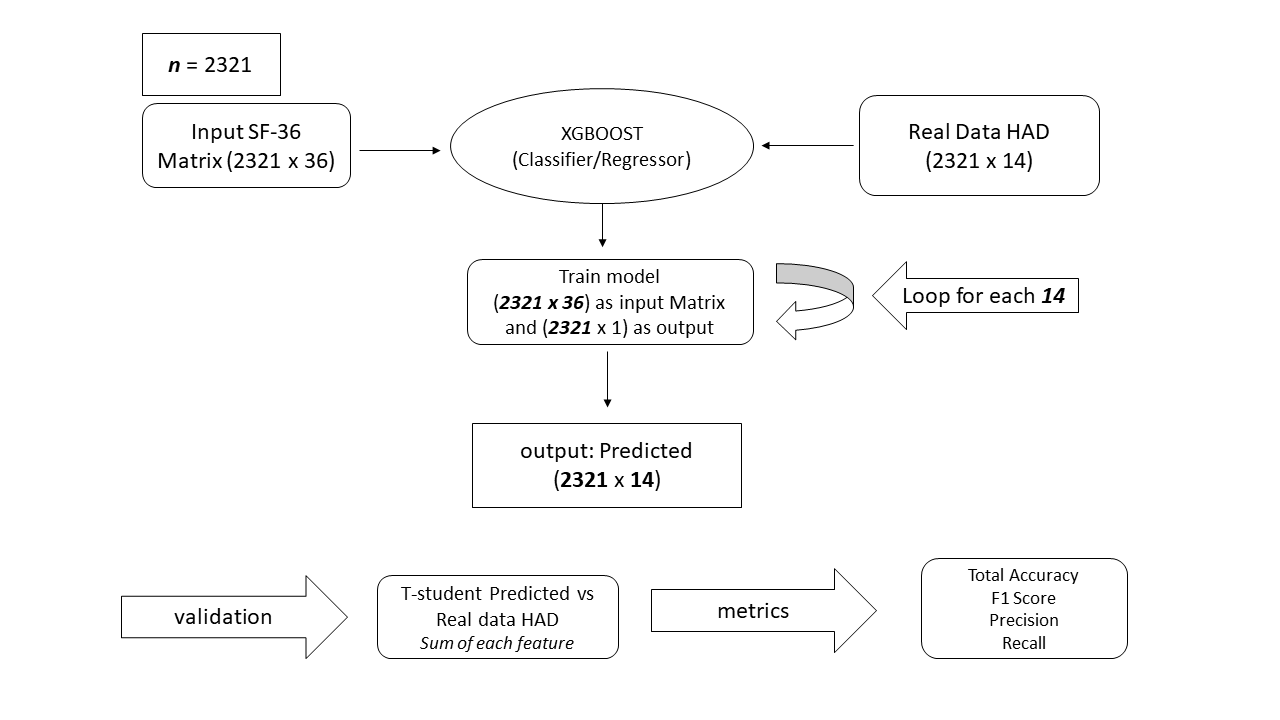


| **Table S1** Comparison results from XBGoost models. | | | | | | |
| --- | --- | --- | --- | --- | --- | --- |
| MODELS | Accuracy | Precision | Recall | F1-Score | Mean Error | t-student |
| XGBoost Regressor | 0.39 | 0.41 | 0.36 | 0.42 | 32.50 % | 1.6e-06 |
| XGBoost Classifier | 0.51 | 0.53 | 0.47 | 0.49 | -3.16 % | 0.60 |

*Total connections have been 32,494 (2321 registers x 14 questions HAD questionnaire) and “1” and “2” answers are 67.25 % from the total. The model tends to reduce the mean error, so the model predicted 70% more “1” than real and rare predicted “3”.*

| **Table S2**. Keras Classifier comparison table from results. | | | | | | |
| --- | --- | --- | --- | --- | --- | --- |
| MODELS | Accuracy | Precision | Recall | F1 Score | Mean Error | t-student |
| K. Classifier with Weights | 0.70 | 0.69 | 0.72 | 0.70 | -1.35 % | 0.79 |
| K. Classifier | 0.50 | 0.51 | 0.46 | 0.47 | -1.80 % | 0.74 |

| **Table S3.** Keras with no weights model results | | | | |
| --- | --- | --- | --- | --- |
| Answers | precision | recall | f1-score | support |
| 0 | 0.00 | 0.00 | 0.00 | 66 |
| 1 | 0.69 | 0.65 | 0.67 | 886 |
| 2 | 0.51 | 0.59 | 0.55 | 807 |
| 3 | 0.61 | 0.60 | 0.61 | 562 |
| accuracy |  |  | 0.60 | 2321 |
| macro avg | 0.45 | 0.46 | 0.46 | 2321 |
| Weighted avg | 0.59 | 0.60 | 0.59 | 2321 |
